# Unravelling socio-motor biomarkers in schizophrenia

**DOI:** 10.1038/s41537-016-0009-x

**Published:** 2017-02-01

**Authors:** Piotr Słowiński, Francesco Alderisio, Chao Zhai, Yuan Shen, Peter Tino, Catherine Bortolon, Delphine Capdevielle, Laura Cohen, Mahdi Khoramshahi, Aude Billard, Robin Salesse, Mathieu Gueugnon, Ludovic Marin, Benoit G. Bardy, Mario di Bernardo, Stephane Raffard, Krasimira Tsaneva-Atanasova

**Affiliations:** 1 Department of Mathematics, College of Engineering, Mathematics and Physical Sciences, University of Exeter, Exeter, EX4 4QF UK; 2 Department of Engineering Mathematics, University of Bristol, Merchant Venturers’ Building, Exeter, BS8 1UB UK; 3 School of Computer Science, University of Birmingham, Edgbaston, Birmingham, B15 2TT UK; 4 University Department of Adult Psychiatry, Hôpital de la Colombière, CHU Montpellier, Montpellier-1 University, Montpellier, France; 5 INSERM U-1061, Montpellier, France; 6 LASA Laboratory, School of Engineering, Ecole Polytechnique Federale de Lausanne—EPFL, Station 9, Lausanne, 1015 Switzerland; 7 EuroMov, Montpellier University, 700 Avenue du Pic Saint-Loup, Montpellier, 34090 France; 8 Institut Universitaire de France, Paris, France; 9 Department of Electrical Engineering and Information Technology, University of Naples Federico II, Naples, 80125 Italy; 10 Epsylon Laboratory Dynamic of Human Abilities & Health Behaviors, Montpellier-3 University, Montpellier, France; 11 EPSRC Centre for Predictive Modelling in Healthcare, University of Exeter, Exeter, EX4 4QJ UK

## Abstract

We present novel, low-cost and non-invasive potential diagnostic biomarkers of schizophrenia. They are based on the ‘mirror-game’, a coordination task in which two partners are asked to mimic each other’s hand movements. In particular, we use the patient’s solo movement, recorded in the absence of a partner, and motion recorded during interaction with an artificial agent, a computer avatar or a humanoid robot. In order to discriminate between the patients and controls, we employ statistical learning techniques, which we apply to nonverbal synchrony and neuromotor features derived from the participants’ movement data. The proposed classifier has 93% accuracy and 100% specificity. Our results provide evidence that statistical learning techniques, nonverbal movement coordination and neuromotor characteristics could form the foundation of decision support tools aiding clinicians in cases of diagnostic uncertainty.

## Mirror game test could detect schizophrenia

A new test of movement and social interaction could detect markers of schizophrenia, and help to diagnose and manage the condition. In an effort to establish reliable indicators of schizophrenia, Piotr Slowinski at the University of Exeter, UK and colleagues developed a test that could detect deficits in movement and social interactions, both characteristics of the disorder. They asked people to perform movements alone, and to mirror the movements of a computer avatar or a humanoid robot. Automated analysis of the movements allowed to distinguish people with schizophrenia from healthy participants with accuracy and specificity slightly better than clinical interviews and comparable to test based on much more expensive neuroimaging methods. The technique could help with diagnosis of schizophrenia and to monitor patients’ responses to treatment, but needs to be tested in clinical trials before being applied in clincal practice.

## Introduction

Schizophrenia is a neurodevelopmental disorder that appears to originate from disruptions in brain development caused by both genetic and environmental factors.^1–3^ With mean lifetime prevalence just below 1%, schizophrenia ranks among the most substantial causes of death worldwide^4^ and is considered as one of the top 25 leading causes of disability.^5^ Due to the high prevalence and lack of entirely satisfactory treatments, a significant research effort has been focused on developing methods for early diagnosis and designing effective preventive interventions.^3, 6^

As defined by the National Institute of Health working group, a biomarker ‘is a characteristic that is objectively measured and evaluated as an indicator of normal biological processes, pathogenic processes, or pharmacologic responses to an intervention’.^7^ Biomarkers can thus play a critical role in performing a diagnostic procedure (diagnostic biomarkers), predicting diagnostic conversion (predictive biomarkers), as well as predicting and monitoring clinical response to psychosocial or pharmacological treatments (prognostic biomarkers).^8–10^ In the last 20 years, increasingly sensitive and sophisticated assessment tools have been developed, and used to identify multiple environmental, neural, molecular and genetic variables as risk factors, and potential biomarkers for schizophrenia.^10–12^ Nevertheless, valid biomarkers for this condition are still lacking or being evaluated.^8^

Long-term persistence of motor and movement impairments in schizophrenia patients have been known since its early description by Bleuler,^13^ and through the years, multiple indicators of schizophrenia based on neuromotor characteristics and variables have been proposed.^14–16^ Many of them are based on motor and socio-motor impairments, which encompass both neurological soft signs (NSS)^17^ and other movement deficits.^18–24^ More specifically, schizophrenia is associated with psychomotor slowing,^21, 25^ characterised by larger reaction times as well as deficits in motor coordination, poor performance in complex motor tasks^20, 26–28^ and weaker interpersonal coordination.^29, 30^ Another class of motor-related abnormalities observed in schizophrenia patients are extrapyramidal symptoms and signs that include: dystonia (continuous spasms and muscle contractions), akathisia (motor restlessness), dyskinesia (irregular, jerky movements), and parkinsonism characterised by rigidity, bradykinesia (slowness of movement) and hypokinesia (decreased bodily movement).^26, 31^ The above mentioned motor abnormalities contribute to the deficits in nonverbal behaviours and in nonverbal synchrony that have been observed in the structured and unstructured social interactions with schizophrenia patients,^22, 29, 32^ which together with deficits in facial behaviour^33^ lead to patients’ social-cognitive impairments and low social competence.

There is now clear evidence that neuromotor abnormalities are present before the onset of the disease and constitute important indicator of schizophrenia.^14, 15^ In fact, NSS such as poor coordination, clumsiness and unfamiliar movements or mannerisms have been recognised as possibly the most common motor abnormalities among children who later developed schizophrenia.^16^ Typically, these symptoms are assessed by a highly skilled clinician during a structured neurological interview, that includes different observational and evaluator-dependent motor-response tests, e.g. finger-thumb opposition (touching fingers in turn with the thumb), bringing the finger to the nose (with eyes closed), diadochokinesis (test of ability to make antagonistic movements in quick succession), rapid manipulation of matchsticks or pegboards, finger following, copying of simple geometric figures.^18, 27^ Furthermore, it is noteworthy that although schizophrenia patients are known to have deficits in interpersonal interactions,^22, 29, 32^ most of the motor assessment tasks do not take into account the motor abnormalities that are present during an interaction with another person.^23, 29, 30^ This is, however, changing and newly proposed measures of extrapyramidal symptoms and NSS are data-driven and are often based on interpersonal interactions, e.g. indices based on digitised hand-writing^26^ or on video recordings of face-to-face interaction with schizophrenia patients.^22, 32^

**Fig. 1:**
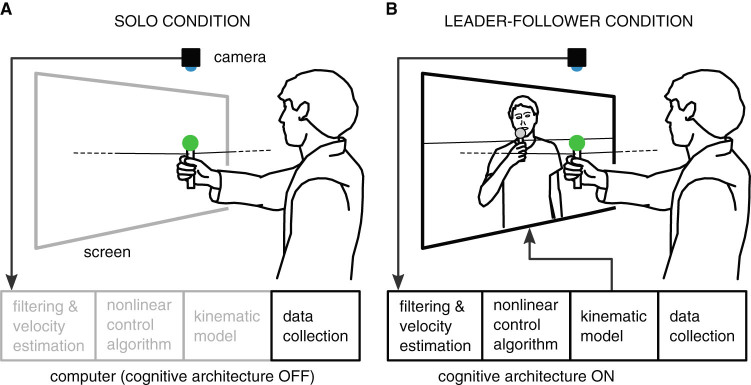
Illustration of the main experimental set-up and of the experimental conditions. **a** Solo condition of the mirror game, hand movement of the participant along a line is recorded on a computer. **b** In the leader–follower condition participant is following motion of a computer avatar that is displayed on the screen

In our study, we employ neuromotor characteristics extracted from participants’ motion recorded during two different conditions of a simplified ‘mirror-game scenario’; a joint-action task, considered currently a paradigm for studying interpersonal coordination,^34, 35^ in which two participants are asked to mimic each other’s movements. In the solo condition, each participant is asked to move in a natural, interesting manner in the absence of a partner (Fig. 1a), while in the leader–follower condition the participant is instructed to follow as accurately as possible the motion of the other player acting as the leader (Fig. 1b). We demonstrate that movement properties measured in the solo condition allow for quantification of extrapyramidal symptoms in a manner similar to what has been shown in previous literature.^26^ In contrast coordination measures extracted from the leader–follower condition capture changes related to psychomotor slowing^21, 25^ and deficits in interpersonal synchrony.^22, 29, 32^ In so doing, the two distinct conditions of the mirror game make it possible to capture and quantify complementary characteristics of intra and interpersonal motor behaviour.

To quantify participants’ movements, we develop a data-driven, objective methodology based on different aspects of the recorded motion. Furthermore, we use personalised artificial agents (computer avatars and a humanoid robot) acting as leaders in the leader–follower condition.^35, 36^ Notably, this allows us to achieve discrimination on the level of individuals, in line with previous work.^12, 15^

The artificial agents used in our experiments are driven by a cognitive architecture based on feedback-control theory modelling of perception–action behaviour,^37–39^ which was developed within the scope of the European Alterego project (http://www.euromov.eu/alterego/). Importantly, in addition to generating a leader’s motion with desired level of difficulty for each individual player, the cognitive architecture allows for a bi-directional feedback between the participant and the artificial agent. Such feedback is one of the aspects of the complex social interactions that constitute the basis of the socio-motor coordination in joint-action tasks.^40^ In fact, the bi-directionality of the feedback is a unique feature of the proposed methodology, which differentiates it from other motor assessment tools. Finally, interaction with artificial agents contributes to the objective nature of the proposed methodology that is practically impossible to achieve in classical interactions between human subjects. Namely, artificial agents allow us to eliminate effects of negative attitudes that are often present in interactions between non-clinical individuals and patients.^41, 42^

## Results

The two main results of our study are the classification methodology and the set of unbiased, data-driven neuromotor markers of schizophrenia, which are extracted from recordings of participants’ spontaneous hand motion^35^ and their movement during interaction with an artificial agent. We begin by presenting the neuromotor markers (features) extracted from the data recorded in solo condition and the results of a classification based on them. Next, we present neuromotor markers (features) extracted from data recorded in the leader–follower condition of the mirror game in which the participant is instructed to follow the motion of a computer avatar,^35^ complimented by the corresponding classification results. We, then, validate the neuromotor markers (features) extracted from data recorded in the leader–follower condition as well as the classification pipeline using data collected in an independent experiment with a humanoid iCub robot involving different group of patients and control subjects. Finally, we demonstrate that the classification is significantly improved by applying majority rule to combine the results obtained for the solo and leader–follower conditions. We show, additionally, that the classification based on the proposed biomarkers is complementary to classifier based on the NSS evaluated by an expert clinician. Details of the experimental protocols, features and of the classification algorithm can be found in Materials and Methods.

### Classification—solo condition

In the solo condition, we recorded each participant’s spontaneous movement limited only by the physical set-up of the recording equipment, see Materials and Methods for details. The motivation for using features of the solo movements as biomarkers stems from our recent finding that individual people have unique, time-persistent motor signatures^35^ and from research showing that fine-motor skills are affected in schizophrenia, e.g., person’s handwriting.^26^

**Fig. 2:**
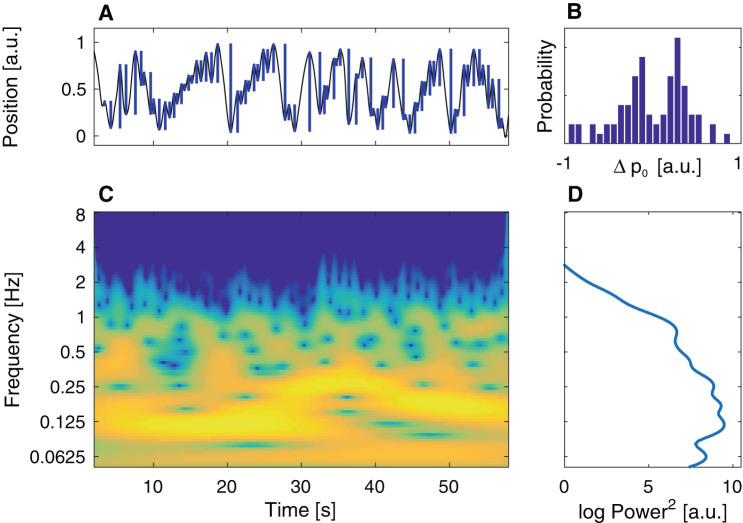
Features based on the solo movement. **a** Position time trace from a solo recording, blue bars indicate lengths of movement segments in the left and right direction *ΔP*_0_. **b** Histogram of the values of *ΔP*_0_ from panel **a**. **c** Time-frequency plot of the wavelet transform of the time series from panel **a**. Colour indicates power for given frequency at a given time. **d** GWS computed as mean (over time) of the signal representation in panel (**c**)

In order to capture nuances of the solo movement (Fig. 2a), we propose three features for its classification. Two of the features are derived from a stochastic model of hand motion, which follows the integrated human movement framework;^43^ for a description of the model see the Supplementary Note. Specifically, the two features are: *ΔP*_0_, histograms (bar plots) of lengths of movement segments in the left and right direction (Fig. 2b), and distributions of coefficients of the stochastic model of the hand motion. The third feature is the global wavelet spectrum (GWS) (Fig. 2d) that captures frequencies of different oscillations observed in the motion.^44^

The results of the data classification from the solo condition can be found in Table 1 (row Solo).

**Table 1:** Results of classification based on different features

Data	Ctrls/Pts	TN	FP	TP	FN	Accuracy	Sensitivity	Specificity	Precision
Solo (S)^a^	29/30	28	1	27	3	0.9322	0.9	0.9655	0.9642
Leader–follower (L–F)^b^	29/30	28	1	17	13	0.7627	0.5667	0.9655	0.9444
**Majority: S and L–F** ^c^	29/30	**29**	**0**	26	4	**0.9322**	0.8667	**1**	**1**
Validation: iCub L–F^d^	22/22	21	1	17	5	0.8636	0.7727	0.9545	0.9444
NSS^e^	26/30	25	1	24	6	0.8750	0.8000	0.9615	0.9600

Definitions of classification measures can be found in Materials and Methods

*TP* true positive, *FP* false positive, *TN* true negative, *FN* false negative

^a^ Results of classification based on Solo condition features. Majority rule applied to results of classification based on the separate features extracted from the solo data. Results of classification using the separate features can be found in Supplementary materials—Table S1

^b^ Results of classification based on the leader–follower condition in the avatar experiment. Majority rule applied to results of classification based on the separate features extracted from the leader–follower data. Results of classification using the separate features can be found in Supplementary materials–Table S2

^c^ Results of majority rule applied to results of classification of Solo condition and leader–follower condition. Majority rule applied to results of classification based on all the separate features presented in the Supplementary Materials—Tables S1 and S2

^d^ Results of classification based on the leader–follower condition recorded in the iCub experiment. Majority rule applied to results of classification using the separate features extracted from the leader–follower data. Results of classification using the individual features can be found in Supplementary materials—Table S3

^e^ Results of classification based on the NSS. Best classification was achieved using: gait—arms swinging, salivation and arms dropping as defined in ref. 17 (linear discriminant classifier with leave-one-out validation). Exactly the same results (in terms of participants classified as patients) were obtained for several other combinations of features

### Classification—leader–follower condition

**Fig. 3:**
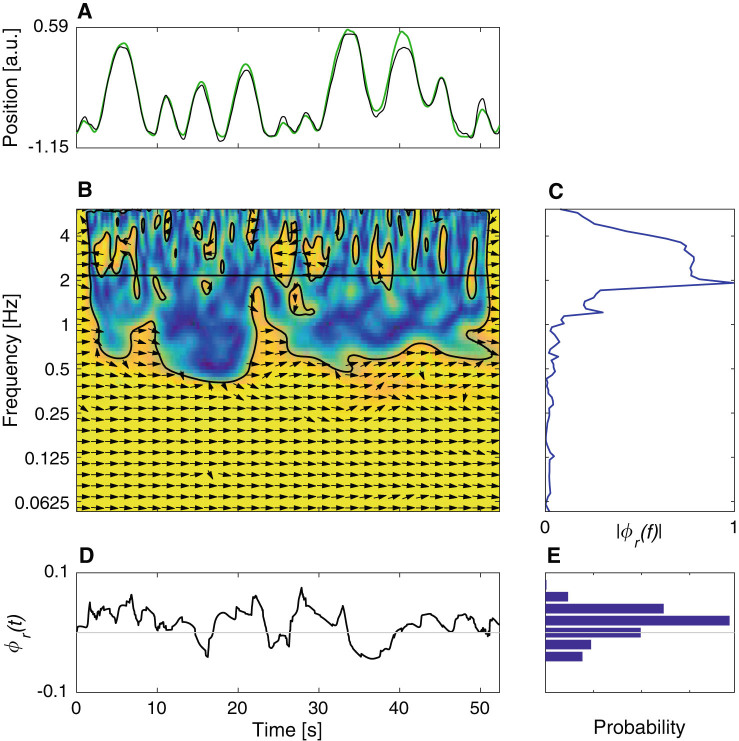
Features based on the artificial-agent leader—human follower interaction. **a** Position time traces of a leader (*black*) and follower (*green*). **b** Time-frequency plot of the cross-wavelet coherence.^54^ Arrows indicate relative phase (3 o’clock indicates in-phase). Colour indicates degree of "overlap" between wavelet spectra of the two time series. **c** *|ϕ*_*r*_ *(f)|*—distribution of absolute phase lag over frequencies, computed as the absolute value of a circular mean (over time) of relative phase angles from the regions encircled by the black lines in panel **b**. **d** Estimate of the relative phase *ϕ*_*r*_ *(t)*, computed as circular mean (over frequencies) of the relative phase angles from panel **b** for frequencies lower than 2 Hz, y-axis in radians. **e** Histogram of the relative phase *ϕ*_*r*_ *(t)* from panel **d**

A number of studies have shown that schizophrenia patients behave differently during social interactions.^22, 23, 29, 30, 32^ To capture some basic aspects of the differences in the socio-motor coordination in data collected in the leader–follower condition, we used features derived from the relative phase (Fig. 3b), which captures the time lag between two participants and is a well-established method for analysing inter-personal coordination.^35, 45, 46^ The two features are: a distribution of lags between phases of oscillations with different frequencies observed in the movements of the leader and follower *|ϕ*_*r*_ *(f)|* (Fig. 3c), and a histogram of the relative phase during interaction, *ϕ*_*r*_ *(t)* (Fig. 3e), which describes changing time lag between movements of the leader and the follower.

The features selected for analysis of the leader–follower condition provide information complementary to that based on solo condition, as we anticipate that the properties of the motion of participants following an artificial agent would be affected mostly by reduced reaction times, deficits in motor coordination and nonverbal synchrony, and to a smaller extent by some of the extrapyramidal symptoms and signs.

The results of the classification based on the leader–follower condition can be found in Table 1 (row Leader–follower). It is worthy of note that, although the accuracy of the classification is lower than in the case of the solo condition, the classifier still has very high specificity and precision (only single participant from the control group was misclassified).

### Classification—majority rule over solo and leader–follower conditions

Next we apply the majority vote rule to all results of classifications based on the separate features extracted from the data collected in the solo and leader–follower conditions. This allows us to achieve unprecedentedly high 93% accuracy, and remarkably completely eliminates false positives; see Table 1 (row Majority: S and L–F) for the other classification measures. This result demonstrates the synergy of using different neuromotor biomarkers for classification. Notably the majority rule significantly increases the classification precision and specificity, as it classifies a participant as a patient only if irregularities are observed in more than half of the analysed neuromotor biomarkers. Thus, in order to eliminate type I errors (false positives), i.e. misclassifying controls as patients, it is beneficial to use as much information as possible and base the final classification on more than one feature.

### Validation and comparison of the avatar and iCub experiments

The data collected in the independent iCub experiment^36^ allows us to validate the classification algorithm as well as compare the performance of our methodology when using two very different types of leader’s movement. We emphasise that for validation we use exactly the same algorithm and features as those used for the classification based on the data collected in the leader–follower condition of the avatar experiment. Results of the validation can be found in the Table 1 (row Validation: iCub L–F). In particular, the results presented in Table 1 (row Validation: iCub L–F) show that the proposed method consistently achieves high specificity and precision. This is even more significant considering the differences in the experimental protocols, see Materials and Methods—Experiments for details.

Furthermore, our results show increase in sensitivity (ratio of correctly classified patients) 57% avatar experiment vs. 77% iCub experiement and accuracy (ratio of correctly classified participants) 76% avatar experiment vs. 86% iCub experiment. We presume that the increase in sensitivity and accuracy is due to the difference in the reference trajectories used in the two experimental conditions. Specifically, the iCub experiment used the same set of reference trajectories for every participant, while in the avatar experiment, the reference trajectory was individualised for every person as it was generated using pre-recorded solo movement data of that person. Furthermore, due to the hardware limitations, the motion of the iCub robot was slower and had shorter range than the motion of the virtual avatar. In our opinion, the observed difference in the discriminative power of classification based on coordination measures might indicate that the slower and less familiar motion of the iCub robot was more difficult to follow for patients than their own more familiar movement. Interestingly, this observation echoes our recent finding that following own movements is an easier task than following a trajectory different from our preferred style of motion.^35^

These observations are further corroborated by the fact that there is only a single false positive error in both cases, i.e., the obtained specificity and precision have effectively the same value, the highest possible value other than 100% (100% is obtained in case of lack of false positive errors). Single false positive error suggests that the type of reference trajectory affected only performance of the patients in the leader follower task. It is possible, that following atypical movement requires more mental effort from patients than controls.^19^ Therefore, in future applications it is reasonable to expect higher discriminative power in the case of classification based on coordination measures if the movement of the leader is atypical and less familiar, e.g. as in our case slower and with shorter physical range.

Finally, we compared if the TP (patients classified as patients) and FN (patients classified as controls) for the leader–follower condition in the two experiments differ with respect to the positive and negative syndrome scale (PANSS) as assessed by an expert clinician. We found that the FN group in the avatar experiment has lower level of negative syndromes (*p* = 0.022 using Mann–Whitney–Wilcoxon U) as well as lower general psychopathology score (*p* = 0.011 using Mann–Whitney–Wilcoxon U) than the TP group; this observation agrees with previously reported results.^47^ Such differences, however, are not observed in the TP and FN groups from the iCub experiment (*p* = 0.082 for negative syndromes and *p* = 0.61 for general psychopathology score; both based on Mann–Whitney–Wilcoxon U). These findings suggest that severity of the symptoms affects the level of coordination when the patients are following familiar motion and further indicate that using atypical movement in the leader–follower condition could be advantageous for classification purposes. We did not find any such correlation for the solo condition of the avatar experiment.

### Classification—comparison of the majority vote rule with NSS

We next compared results of the classification based on the majority vote rule with those obtained from the classification based on the standardised set of NSS,^17^ which were evaluated by an experienced psychiatrist during a neurological examination that was performed before each experiment. Hence, the evaluator was blind to the patient’s mirror game performance. For three participants from the control group, the NSS data was unavailable.

For NSS classification, the same methodology treating each variable in a questionnaire as a feature was employed (algorithm modifications are detailed in Materials and Methods). The best classification (Table 1, row NSS) was achieved using the following features: gait—arms swinging, salivation and arms dropping as defined in^17^ Direct comparison between the results presented in Table 1, demonstrates that classification based on the neuromotor biomarkers performs slightly better than the classification based on the NSS.

### Dependence of the results on the equivalent chlorpromazine dose

Finally, we tested whether the results of our classifications obtained by means of the majority rule and based on the NSS depend on the equivalent chlorpromazine dose of psychotropic medication of the patients. When comparing doses in the groups given by TP and FN (Majority: S and L–F classification) the probability that doses in the two groups are the same is equal to *p* = 0.9714 using Mann–Whitney–Wilcoxon U; for TP and FN (NSS classification) the probability that doses in the two groups are the same is equal to *p* = 0.8849 using Mann–Whitney–Wilcoxon U. All the performed statistical tests confirm that the classification outcomes are unaffected by the dose of medication taken by the patients. This analysis did not include one patient for whom we do not have data.

## Discussion

The method developed in our study uses neuromotor biomarkers based on movement characteristics, fine-motor coordination and visual-motor coordination to discriminate between schizophrenia patients and control subjects on the level of individuals. We have also validated the proposed classification pipeline using data collected in an independent experiment with a humanoid iCub robot.^36^ The proposed biomarkers allow for more accurate classification, in particular with regard to higher specificity and precision compared to existing methods based on neuromotor biomarkers^14, 15, 18^ and are as accurate as some proposed classifiers based on neuroimaging, cognitive, genetic and socio-environmental features.^11, 48^ Furthermore, classification results using the NSS (Table 1, row NSS) demonstrate that statistical learning techniques have potential to form a basis for the development of quantitative clinical decision support tools for analysing data collected during routine neurological examinations. Although, the samples in the individual experiments in our study can be considered small, when considered together they have a sample size (*N* = 109, 52 patients) that is typical for neuroimaging biomarker studies.^11^ Additionally, the fact that our findings have been validated in two independent experiments strengthens our conclusions.

The advantage of using the simplified mirror-game^34, 35^ is that the recorded data can be analysed in an un-biased and quantitative manner, while allowing for a degree of spontaneity of human motion and interaction. Furthermore, the investigated conditions encompass, in a natural way, individual as well as inter-personal aspects of NSS and neuromotor deficits. In particular, the leader–follower task and the bi-directional feedback offered by the cognitive architecture allow to capture some aspects of motor abnormalities that are related to social communication. On the other hand, exploiting artificial agents allows for high degree of control over the experimental conditions, which is practically unachievable in experiments involving interactions with human partners. In particular, interactions with artificial agents are free of negative attitudes and prejudices toward patients, that are often held by non-clinical individuals.^41, 42^ We thus reveal that such objective, highly controllable and at the same time flexible (personalisable) setting, involving aspects of socio-motor interaction, is better suited to test psycho-motor differences between controls and schizophrenia patients than the existing motor assessment tools. The observed lack of 100% accuracy (FN > 0 for all classification results) and the correlation between classification results for the leader–follower condition in the avatar experiment and the severity of the patients’ negative syndromes as well as their general psychopathology score (measured with PANSS) indicate that patients display different degrees of socio-motor impairments. However detailed investigations of the differences between patients’ sub-groups is beyond the scope of this paper.

The main limitation of our study, which also applies to all other studies using biomarkers and classification techniques (including neuroimaging studies), is the fact that the collected data comes from medicated patients. This is important because most of the anti-psychotic drugs have side effects that influence neuromotor behaviour.^3, 16^ These side effects typically include tardive or withdrawal dyskinesia (involuntary or abnormal movements), parkinsonism (tremor, bradykinesia, slowness, rigidity), and akathisia (the feeling of inner restlessness and associated need to be in constant motion, e.g., rocking or leg crossing). Although we do not know to what degree the patients, and hence the results of the current study are affected by the side effects of antipsychotic medications, we have verified that our results do not depend on the equivalent chlorpromazine dose prescribed to individual patients. It is worthy of note that there is a growing body of evidence suggesting that changes in kinematics are inherent to schizophrenia and can be observed before onset of psychosis^14, 15, 18, 21, 27, 31^ as well as in medication-free patients.^17, 21, 25–27^ Moreover, it has been demonstrated that subtle hand motor dysfunctions can be differentiated from drug-induced extrapyramidal dysfunction.^26^ Future research with pre-medication and medication-free participants is needed in order to assess diagnostic potential (diagnostic biomarker) of the proposed methodology.

Furthermore, translation of the presented methods to everyday clinical practice requires clinical trials that would evaluate socio-motor functioning in at risk populations and that would be followed-up over time. We believe that such studies, requiring significant effort, are worthwhile because of advantages of the proposed method. Firstly, the experimental set-up with the computer avatar can be easily placed in a clinical environment. It consists of simple and off-the shelf technology, namely a wide-angle camera connected to a computer with installed cognitive architecture, and is significantly cheaper than any neuroimaging equipment. Secondly, the measurement procedure is quick and non-invasive, and has play-like qualities that make it a potentially attractive diagnostic tool for children. This aspect of our method could become particularly important as early screening is considered a key element in prevention and treatment of schizophrenia.^6, 16, 48^ Finally, considering existing results showing that schizophrenia and social phobic patients have different coordination patterns,^49^ we believe that the proposed method has a differential diagnostic potential. If successfully confirmed in future research, it could then inform preventive interventions that could target not only schizophrenia but also a broader range of mental disorders.^8, 50^

Even though, it is only recently that the motor systems domain has been acknowledged as an important factor that could allow for broader understanding of neural substrates of schizophrenia and other mental disorders,^28^ interpersonal coordination has already been recognised as a potential component of new therapeutic protocols based on social-priming and similarity.^24, 51^ Consequently, interaction with artificial agents could become a part of future therapeutic protocols that would allow for real-time monitoring of therapy progress.

## Materials and methods

Patients were recruited from the University Department of Adult Psychiatry (CHRU Montpellier, France) and fulfilled the Diagnostic and Statistical Manual of Mental Disorders criteria for schizophrenia. Diagnoses were established using the Structured Clinical Interview for DSM–IV-TR (SCID49). All patients received antipsychotic medication. The patients were in the stable phase of the illness according to the current treating psychiatrist and as defined by having no hospitalisations or changes in housing in the month prior to entering the study.

Exclusion criteria were substance dependency other than cannabis or tobacco, substance abuse other than cannabis or alcohol, and co-morbid neurological disorder.

Age and gender-matched healthy participants were recruited from a call for participation on the hospital’s website and local community. They had no lifetime history of any psychosis or affective disorders diagnosis according to the MINI.^52^ Controls with a family member with bipolar or schizophrenia disorders were excluded.

All participants were native French speakers with a minimal reading level validated using the National Adult Reading Test f-NART.^53^

All participants provided written informed consent, prior to the experiment approved by the National Ethics Committee (CPP Sud Mediterannee III, Nımes, France, #2009.07.03ter and ID-RCB-2009-A00513-54) conforming to the Declaration of Helsinki. The methods in the current study were carried out in accordance with the approved guidelines.

NSS were evaluated by experienced clinicians (clinical psychiatrists) trained in them, and blind to the patient’s mirror game performance.^17^

Demographics of the participants in all the experiments can be found in Table 2.

**Table 2:** Social, demographic and clinical information of schizophrenia patients and healthy controls in the avatar and iCub experiments

Avatar experiment	Patients (*N* = 30)		Controls (*N* = 29)		Statistics
	Mean	Min-Max	Mean	Min-Max	
Age (years)	32.5	18–58	30	22–49	*U* = 423.5, *p* = 0.54
Sex (male/female)	25/4		27/3		*χ* ^2^ = 0.2, *p* = 0.65 (Pearson)
iCub experiment	Patients (*N* = 22)		Controls (*N* = 22)		Statistics
	Mean	Min-Max	Mean	Min-Max	
Age (years)	29	21–45	28	19–46	*U* = 218, *p* = 0.57
Sex (male/female)	17/5		15/7		*χ* ^2^ = 0.11, *p* = 0.735 (Yate)
PANSS	iCub (*N* = 22)		Avatar (*N* = 30)		Statistics
	Mean	Min-Max	Mean	Min-Max	
PANSS positive	10.5	7–18	9.4	7–15	*U* = 284.5, *p* = 0.39
PANSS negative	11.36	7–22	15.1	7–33	*U* = 216, *p* = 0.035
PANSS psychopathology	22.9	17–35	27.1	19–38	*U* = 176, *p* = 0.0065
PANSS total	44.77	31–66	51.1	35–75	*U* = 210, *p* = 0.039

*PANSS* positive and negative syndrome scale, *U* Mann–Whitney test, χ^2^ Chi-squared test

All data analysis and modelling was performed in Matlab.

### Experiments

The cognitive architecture used in the experiments uses nonlinear control algorithms coupled with a kinematic model of the arm motion to generate the artificial agent’s movement (Fig. 1b). The artificial agent’s movement is based on a pre-generated trajectory that is modulated, in real-time, in response to the human player’s performance.^37–39^

Experiment with computer avatar (solo condition and leader–follower condition):

- 29 controls and 30 patients.
- Physical movement range 180 cm. In solo and leader–follower conditions, participants were standing in front of a display showing the computer avatar. A horizontal string was mounted in front of the participant. A ball with a small handle was mounted on the string. Participants were instructed to move the ball left and right along the string.
- Solo condition instruction: play the game on your own, create interesting motions and enjoy playing.
- Avatar leader—human follower condition: 12 recordings (30 s.)
- Reference trajectory: participant’s own pre-recorded motion
- Solo condition: 4 recordings (60 s.)
- Solo condition was recorded before (1 trajectory) and between the leader–follower trials (3 trajectories).
- Recorded position data in arbitrary units in range [0,1]; original sampling rate c.a. 40 Hz (interpolated in post processing to exactly 40 Hz). Low-pass filtering with 2 Hz cut-off done using phase preserving Butterworth filter of degree 2.

Experiment with iCub robot (leader–follower condition):

- 22 controls and 22 patients.
- Robot leader—human follower: 15 recordings (60 s.).
- Physical movements range c.a. 30 cm.
- Reference trajectories: 3 slow, 2 fast (all 5 trajectories were slower than typical solo motion). Trajectories were generated using the generative process described in Supplementary Materials—Generative process, and were based on uniform distributions of durations and lengths of movement segments, not on pre-recorded trajectories.
- Recorded position data is in arbitrary units in range [0,1]; original sampling rate 200 Hz. Low-pass filtering with 2 Hz cut-off done using phase preserving Butterworth filter of degree 2. Data was next down-sampled to 20 Hz.

The main differences between the avatar and iCub experimental protocols are:

- Duration of the leader–follower task: 30 s. in the avatar experiment vs. 60 s. in the iCub experiment;
- Type of reference trajectory: participant’s own trajectory (different for each participant) in the avatar experiment vs. generated human-like slow trajectory in the iCub experiment (the same set of trajectories for all the participants);
- Motion of the iCub robot was much slower than of the computer avatar; speed was limited by the robot hardware limitations;
- Physical range of motion: 180 cm in the avatar experiment vs. 30 cm in the iCub experiment; the range was limited by the robot hardware limitations.

### Features

In contrast to most biomarker studies, rather than using indices based on a single number, we propose features that are distributions of values. We are using distributions because they can be quantitatively compared with each other, while preserving significantly more information about a sample than for example its mean. In consequence, this allows for more accurate discrimination between subjects.

### Solo condition

- *ΔP*_0_, distributions (histograms with 51 equally distributed bins) of signed lengths of movement segments, a part of the position trace between two consecutive points were direction of movement changes (Fig. 2b). For analysis the position time-series were concatenated before computing *ΔP*_0_ and their histograms.
- GWS (Fig. 2d). For analysis GWS from all the recorded trials are averaged before being analysed. The wavelet transform is computed using the toolbox described in;^54^ with default parameter settings.
- Distributions (bivariate histograms on a 100 by 100 grid) of coefficients of the stochastic model of hand motion—a feature inspired by learning in the model space approach;^55^ the generative process used to model human movement follows the integrated human movement framework presented in previous work.^43^ For analysis, the position–time series are concatenated before computing the coefficients of generative process. A detailed description of the stochastic model of hand motion can be found in Supplementary Note.

### Leader–follower condition

- Distributions of the absolute phase lag over frequencies, |*ϕ*_*r*_ *(f)|*, computed as the absolute value of a circular mean (over time) of relative phase angles from the significant regions of cross-wavelet coherence (Fig. 3c). For analysis, distributions from all the recorded trials are averaged before being analysed. The cross-wavelet coherence is computed using the toolbox described in;^54^ with default parameter settings.
- Distributions (histogram with 51 equally distributed bins) of the relative phase during interaction, *ϕ*_*r*_ *(t)* (Fig. 3e); for analysis time-series of *ϕ*_*r*_ *(t)* are concatenated before computing histograms. Estimate of the *ϕ*_*r*_ *(t)* is computed as a circular mean (over frequencies) of the relative phase angles from the significant regions of cross-wavelet coherence for frequencies lower than 2 Hz.

Number of bins in the *ΔP*_*0*_, histograms of *ϕ*_*r*_ *(t)* and bivariate histograms of the generative process coefficients are chosen empirically in a manner that assured good representation of the data; results are not sensitive to small changes in number of bins. Number of frequencies over which the *|ϕ*_*r*_ *(f)|* and the GWS depends on the parameter setting for the computations of wavelet transform and cross-wavelet coherence.

As already mentioned, all features are based on combined data collected in a given condition for each individual participant, this ensures the statistically significant discriminative power provided by larger samples or longer time-series.

### Classification algorithm

Below we specify all steps of our classification and illustrate their application in regard to the distribution of absolute phase lag over frequencies, *|ϕ*_*r*_ *(f)|*, computed for the data from the iCub experiment as an example. Since all our features are distributions we use the same procedure for classifying them. In particular our approach is a dissimilarity-based classification method that uses isometrical embedding of dissimilarity data.^56^ Our methodology can be summarised in the following steps:

Step 1

We compute the earth mover’s distances^57, 58^ between all distributions constructed from the data; for applications of the earth mover’s distances in movement science see;^35^ for each feature we obtain one matrix of size 44 × 44 in the iCub experiment and 59 × 59 in the Avatar experiment.

Step 2

We convert the distances matrix (comprising dissimilarities between the participants, based on a particular feature) into points in an abstract geometric space using Multidimensional Scaling (MDS), which is a standard data mining technique.^59^

Step 3

In order to classify the points in the MDS-space obtained in the previous step, we use standard linear and pseudo-quadratic discriminant analysis as implemented in Matlab. We use simpler discriminants, rather than support vector machines, because they are faster and would be easier to implement and use in real clinical practice. Additionally, we found the computational cost of obtaining optimal parameters for finding support vectors to be quite high.

Step 4

In the spirit of successful strategy of selecting regions of interest from the classifiers applied to neuroimaging data,^60^ we next select the subset of dominant dimensions of the MDS-space. In order to find the best subset, we compare the results of the classifications for all possible combinations of sets of up to 6 coordinates out of first 15 dimensions generated with MDS. This step is necessary, since we do not know what the interpretable, and significant for discrimination, correlates are of the dominant MDS-space coordinates.

Step 5

For each set of coordinates, we run classification applying leave-one-out cross-validation scheme. Leave-one-out cross-validation scheme provides a conservative estimate of accuracy in case of a small number of datasets. The scheme corresponds to the situation that we would like to classify a new participant using classifier based on all the available data. We use the leave-one-out cross-validation scheme also because schizophrenia is a spectrum disease and it is possible that only subpopulation of schizophrenia patients would have symptoms that manifest as neuromotor biomarkers.

Step 6

For each set of the coordinates, we compute the following classification measures:

$$ \mathrm{Accuracy}=\frac{(\mathrm{TP}+\mathrm{TN})}{{\rm{N}}}, $$

$$ \mathrm{Sensitivity}=\frac{{\rm{TP}}}{(\mathrm{TP}+\mathrm{FN})}=\frac{{\rm{TP}}}{{{\rm{N}}}_{\mathrm{Pt},t}}, $$

$$ \mathrm{Specificity}=\frac{{\rm{TN}}}{(\mathrm{TN}+\mathrm{FP})}=\frac{{\rm{TN}}}{{{\rm{N}}}_{\mathrm{Ct},t}}, $$

$$ \mathrm{Precision}=\frac{{\rm{TP}}}{(\mathrm{TP}+\mathrm{FP})}=\frac{{\rm{TP}}}{{{\rm{N}}}_{\mathrm{Pt},p}}{\rm{.}} $$

Here: *N* is total number of participants, TN is number of true negative (controls classified as controls), FN is number of false negative (patients classified as controls), FP is number of false positive (controls classified as patients), TP is number of true positive (patients classified as patients), *N*_Ct,t_ is number of true controls, *N*_Pt,t_ is number of true patients and *N*_Pt,p_ is number of predicted patients. Precision is also known as positive predictive power/value (PPP/PPV).

Step 7

The results of our classification methodology are determined on the basis of the set of the coordinates that give highest accuracy. If two sets of coordinates have the same accuracy, we choose the one with higher precision. If more than one set of coordinates have the same accuracy and precision, we use all of them as classifiers and apply majority rule to their outcomes. Additionally, we compare results obtained with linear and pseudo-quadratic discriminant and choose the one with highest accuracy. In cases when the accuracy is the same for both discriminants, we choose the one with higher precision.

### Majority rule

Majority rule means that we classify a participant as a patient only if s/he is indicated as a patient by results of more than half of classifications based on separate features, e.g., 2 out of 2, 2 out of 3, 3 out of 4, 3 out of 5, etc.

### Illustrative example of the MDS-space and of the Step 7 of the classification algorithm

**Fig. 4:**
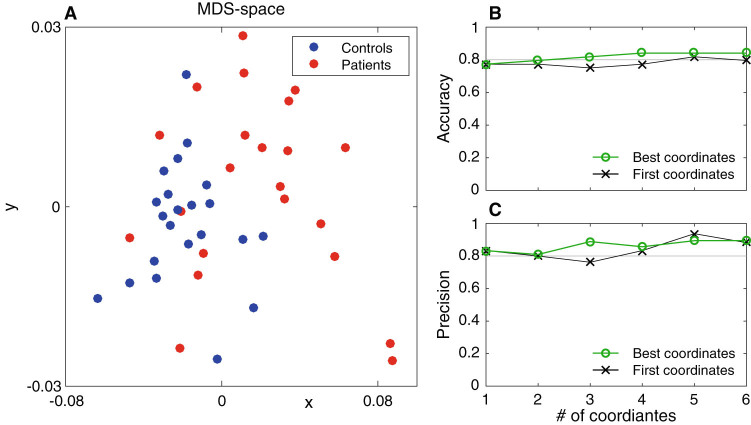
Illustrative example of the MDS-space and of the Step 7 of the classification algorithm. **a** Two first coordinates of the MDS-space computed from the EMDs between distributions of absolute phase lag over frequencies |*ϕ*_*r*_ *(f)|*. *Red dots* indicate patients while *blue dots* indicate controls. Corresponding classification measures are: accuracy = 0.7727, sensitivity = 0.7272, specificity = 0.8181, precision = 0.8000. Panel **b** shows accuracy and **c** precision of classification as a function of number of the coordinates. *Black crosses* show result of classification using the first *n* dominant coordinates. *Green circles* show result of the best classification using the set of *n* coordinates out of 15 dominant coordinates

Figure 4a shows an example representation of the 44 participants using first two dominant dimensions of the MDS-space based on distances between *|ϕ*_*r*_ *(f)|* computed for the data from the iCub experiment. Each point corresponds to a participant, red dots indicate patients and blue controls. The points corresponding to patients and controls occupy two different regions in the MDS-space (Fig. 4a).

We next compare results of classification using different sets of coordinates of the MDS-space based on the EMDs between *|ϕ*_*r*_ *(f)|* distributions (Fig. 4b, c). Even though, the results are already satisfying for the classifiers based on the first *n* dominant coordinates of the MDS-space (*black crosses*—first coordinates), we notice that the accuracy does not increase monotonically, suggesting that the first *n* coordinates are not the optimal set for creating a classifier. In order to validate this observation further, we analyse results of the classification using the sets of *n* optimal coordinates chosen on the basis of the accuracy criterion (*green circles*—Best coordinates). In particular, we observe that for the best coordinates, the accuracy of the classification increases monotonically with the number of coordinates *n*, also the final accuracy when using the set of 6 best coordinates is higher than one achieved using the first 6 dominant coordinates.

### Modifications of the algorithm for classification based on questionnaires

For classification of the questionnaires data we start from Step 4 of the above algorithm, i.e. we treat the values in the questionnaires as coordinates in an abstract geometric space. In the case of the avatar experiment we have 84 NSS variables, we reduced their number by choosing 15 with the lowest *p*-value (<0.3) of the Mann–Whitney–Wilcoxon U test when comparing the value of the variable for the two groups. The 15 selected NNS variables are: gait—left arm swinging, gait—both arms swinging, gait—right arm swinging, fist edge-palm—left hand, apraxia, tandem walk (heel-to-toe), shoulder shaking, right hand (lateral preference), fist edge-palm—sum, shoulder shaking—left shoulder, salivation, arm dropping—left arm, arm dropping—right arm, arm dropping—sum, elbow rigidity—sum. In the case of the iCub experiment we have only 10 variables, which allows us to test all of their possible combinations (see results in Supplementary materials—Table S4). After selecting the variables we continue with Steps 5, 6 and 7.

### Data and materials availability

Fully anonymised data is available upon request.

## Electronic supplementary material

Supplementary Information

## References

[1] Howes OD, Murray RM (2013). Schizophrenia: an integrated sociodevelopmental-cognitive model. Lancet.

[2] Sullivan PF (2012). Puzzling over schizophrenia: Schizophrenia as a pathway disease. Nat. Med..

[3] Kahn RS (2015). Schizophrenia. Nat. Rev. Dis. Primers.

[4] Walker E, McGee RE, Druss BG (2015). Mortality in mental disorders and global disease burden implications: a systematic review and meta-analysis. JAMA Psychiatry.

[5] Global Burden of Disease Study (2013). 2013 Collaborators. Global, regional, and national incidence, prevalence, and years lived with disability for 301 acute and chronic diseases and injuries in 188 countries, 1990–2013: a systematic analysis for the Global Burden of Disease Study 2013. Lancet.

[6] Solis M (2014). Prevention: before the break. Nature.

[7] Biomarkers Definitions Working Group (2001). Biomarkers and surrogate endpoints: preferred definitions and conceptual framework. Clin. Pharmacol. Ther..

[8] Weickert CS, Weickert TW, Pillai A, Buckley PF (2013). Biomarkers in Schizophrenia: a brief conceptual consideration. Dis. Markers.

[9] FDA. *Guidance for Industry and FDA Staff—qualification process for drug development tools.* (FDA, 2014).

[10] Goff DC (2016). Biomarkers for drug development in early psychosis: Current issues and promising directions. Eur. Neuropsychopharmacol..

[11] Orrù G, Pettersson-Yeo W, Marquand AF, Sartori G, Mechelli A (2012). Using Support Vector Machine to identify imaging biomarkers of neurological and psychiatric disease: A critical review. Neurosci. Biobehav. Rev..

[12] Chan MK (2015). Development of a blood-based molecular biomarker test for identification of schizophrenia before disease onset. Transl. Psychiatry.

[13] Bleuler E (1950). Dementia Praecox or the group of Schizophrenias.

[14] Hans SL (1999). Neurobehavioral deficits at adolescence in children at risk for schizophrenia: The jerusalem infant development study. Arch. Gen. Psychiatry.

[15] Erlenmeyer-Kimling L (2000). Attention, memory, and motor skills as childhood predictors of schizophrenia-related Psychoses: the New York High-Risk Project. Am. J. Psychiatry..

[16] Liu CH, Keshavan MS, Tronick E, Seidman LJ (2015). Perinatal risks and childhood premorbid indicators of later psychosis: next steps for early psychosocial interventions. Schizophr. Bull..

[17] Krebs M-O, Gut-Fayand A, Bourdel M-C, Dischamp J, Olié J-P (2000). Validation and factorial structure of a standardized neurological examination assessing neurological soft signs in schizophrenia. Schizophr. Res..

[18] Marcus J, Hans SL, Lewow E, Wilkinson L, Burack CM (1985). Neurological findings in high-risk children: childhood assessment and 5-year followup. Schizophr. Bull..

[19] Holthausen EAE, Wiersma D, Knegtering RH, Van den Bosch RJ (1999). Psychopathology and cognition in schizophrenia spectrum disorders: the role of depressive symptoms. Schizophr. Res..

[20] Dazzan P, Murray RM (2002). Neurological soft signs in first-episode psychosis: a systematic review. Br. J. Psychiatry.

[21] Morrens M, Hulstijn W, Sabbe B (2007). Psychomotor slowing in schizophrenia. Schizophr. Bull..

[22] Lavelle M, Healey PGT, McCabe R (2013). Is nonverbal communication disrupted in interactions involving patients with schizophrenia?. Schizophr. Bull..

[23] Capdevielle D (2015). Social motor coordination in schizophrenia patients: from impairment to rehabilitation. Eur. Psychiatry.

[24] Raffard S (2015). Social priming enhances interpersonal synchronization and feeling of connectedness towards schizophrenia patients. Sci. Rep..

[25] Vinogradov S, Poole JH, Willis-Shore J, Ober BA, Shenaut GK (1998). Slower and more variable reaction times in schizophrenia: what do they signify?. Schizophr. Res..

[26] Henkel V (2004). Kinematical analysis of motor function in schizophrenic patients: a possibility to separate negative symptoms from extrapyramidal dysfunction induced by neuroleptics?. Pharmacopsychiatry.

[27] Mittal VA, Neumann C, Saczawa M, Walker EF (2008). Longitudinal progression of movement abnormalities in relation to psychotic symptoms in adolescents at high risk of schizophrenia. Arch. Gen. Psychiatry..

[28] Bernard JA, Mittal VA (2015). Updating the research domain criteria: the utility of a motor dimension. Psychol. Med..

[29] Varlet M (2012). Impairments of social motor coordination in schizophrenia. PLoS One.

[30] Varlet M (2014). Difficulty leading interpersonal coordination: towards an embodied signature of social anxiety disorder. Front. Behav. Neurosci..

[31] Honer WG, Kopala LC, Rabinowitz J (2005). Extrapyramidal symptoms and signs in first-episode, antipsychotic exposed and non-exposed patients with schizophrenia or related psychotic illness. J. Psychopharmacol..

[32] Kupper Z, Ramseyer F, Hoffmann H, Tschacher W (2016). Nonverbal synchrony in social interactions of patients with schizophrenia indicates socio-communicative deficits. PLoS One.

[33] Brüne M, Abdel-Hamid M, Sonntag C, Lehmkämper C, Langdon R (2009). Linking social cognition with social interaction: non-verbal expressivity, social competence and “mentalising” in patients with schizophrenia spectrum disorders. Behav. Brain Funct..

[34] Noy L, Dekel E, Alon U (2011). The mirror game as a paradigm for studying the dynamics of two people improvising motion together. Proc. Natl. Acad. Sci..

[35] Słowiński P (2016). Dynamic similarity promotes interpersonal coordination in joint action. J. R. Soc. Interface.

[36] Raffard, S., et al. Humanoid robots versus humans: how is emotional valence of facial expressions recognized by individuals with schizophrenia? An exploratory study. *Schizophr. Res.* **176**, 506–513 (2016). 10.1016/j.schres.2016.06.001 27293136

[37] Zhai C, Alderisio F, Słowiński P, Tsaneva-Atanasova K, di Bernardo M (2016). Design of a Virtual player for joint improvisation with humans in the mirror game. PLoS One.

[38] Zhai, C., Alderisio, F., Tsaneva-Atanasova, K. & di Bernardo, M. *A novel **cognitive architecture for a human-like virtual player in the mirror game*. *I**n 2014 IEEE International Conference on Systems, Man, and Cybernetics (SMC)* 754–759 (IEEE, 2014).

[39] Zhai, C., Alderisio, F., Tsaneva-Atanasova, K. & di Bernardo, M. *A model predictive approach to control the motion of a virtual player in the mirror game*. *In 2015 54th IEEE Conference on Decision and Control (CDC)* 3175–3180 (IEEE, 2015).

[40] Ouwehand PW, Peper CE (2015). Does interpersonal movement synchronization differ from synchronization with a moving object?. Neurosci. Lett..

[41] Kasow ZM, Weisskirch RS (2010). Differences in attributions of mental illness and social distance for portrayals of four mental disorders. Psychol. Rep..

[42] West K, Hewstone M, Lolliot S (2014). Intergroup contact and prejudice against people with schizophrenia. J. Soc. Psychol..

[43] Viviani P, Flash T (1995). Minimum-jerk, two-thirds power law, and isochrony: converging approaches to movement planning. J. Exp. Psychol. Hum. Percept. Perform..

[44] Issartel J, Marin L, Gaillot P, Bardainne T, Cadopi M (2006). A practical guide to time-frequency analysis in the study of human motor behavior: the contribution of wavelet transform. J. Mot. Behav..

[45] Issartel, J., Gaillot, P., Bardainne, T. & Marin, L. The relevance of the cross-wavelet transform in the analysis of human interaction—a tutorial. *Front. Psychol.* **5**, 1566 (2015). 10.3389/fpsyg.2014.01566 PMC4288242 25620949

[46] Schmidt, R., Nie, L., Franco, A. & Richardson, M.J. Bodily synchronization underlying joke telling. *Front. Hum. Neurosci.* **8**, 633 (2014). 10.3389/fnhum.2014.00633 PMC4133755 25177287

[47] Bernard JA (2014). Cerebellar networks in individuals at ultra high-risk of psychosis: impact on postural sway and symptom severity. Hum. Brain Mapp..

[48] Cannon TD, Cadenhead K, Cornblatt B (2008). Prediction of psychosis in youth at high clinical risk: a multisite longitudinal study in north america. Arch. Gen. Psychiatry..

[49] Capdevielle D (2013). 1283—Social motor coordinations: a study with schizophrenia and social phobic patients. Eur. Psychiatry.

[50] Sommer IE (2016). Early interventions in risk groups for schizophrenia: what are we waiting for?. NPJ Schizophr..

[51] Del-Monte J (2014). Social priming increases nonverbal expressive behaviors in schizophrenia. PLoS One.

[52] Sheehan, D. V. *et al*. The development and validation of a structured diagnostic psychiatric interview for DSM-IV and ICD-10. Mini-Int. Neuropsychiatr. Interv. **59**, 22–33 (1998). 9881538

[53] Mackinnon A, Mulligan R (2005). The estimation of premorbid intelligence levels in French speakers. Encephale.

[54] Grinsted A, Moore JC, Jevrejeva S (2004). Application of the cross wavelet transform and wavelet coherence to geophysical time series. Nonlinear Process. Geophys..

[55] Shen, Y., Tino, P. & Tsaneva-Atanasova, K. A Classification Framework for Partially Observed Dynamical Systems. Eprint at arXiv:1607.02085 (2016). 10.1103/PhysRevE.95.043303 28505824

[56] Paclík P, Duin RPW (2003). Dissimilarity-based classification of spectra: computational issues. Real-Time Imaging.

[57] Muskulus M, Verduyn-Lunel S (2011). Wasserstein distances in the analysis of time series and dynamical systems. Physica D.

[58] Peyre, G. The numerical tours of signal processing. *IEEE Computing in Science and Engineering*, **13**, 94–97 (2011).

[59] Borg I, Groenen PJF (2005). Modern multidimensional scaling: Theory and applications.

[60] Chu C, Hsu A-L, Chou K-H, Bandettini P, Lin C (2012). Does feature selection improve classification accuracy? Impact of sample size and feature selection on classification using anatomical magnetic resonance images. Neuroimage.

