# Supplementary material for: Unravelling socio-motor biomarkers in schizophrenia
Source: NPJ Schizophr. 2017 Feb 1;3:8. doi: 10.1038/s41537-016-0009-x (PMC5441525; doi:10.1038/s41537-016-0009-x)
Supplement: Supplementary file 1 — Supplementary Information [file 41537_2016_9_MOESM1_ESM.docx]

**Supplementary Materials**

*Table S1 Results of classification of based on the features extracted from the solo data from the avatar experiment, 30 patients and 29 controls. Features: (c_4_, c_3_), (c_4_, c_2_), (c_3_, c_2_) – 2D histograms of pairs coefficients of the generative process; $\Delta p0$ – histograms of signed lengths of movement segments; GWS – global wavelet spectrum; Maj – majority over (c_4_, c_3_), (c_4_, c_2_), (c_3_, c_2_), $\Delta p0$, GWS.*

| Feature | Ctrls/Pts | TN | FP | TP | FN | Accuracy | Sensitivity | Specificity | Precision |
| --- | --- | --- | --- | --- | --- | --- | --- | --- | --- |
| (c_4_, c_3_) | 29/30 | 23 | 6 | 21 | 9 | 0.7458 | 0.7000 | 0.7931 | 0.7778 |
| (c_4_, c_2_) | 29/30 | 25 | 4 | 21 | 9 | 0.7797 | 0.7000 | 0.8621 | 0.8400 |
| (c_3_, c_2_) | 29/30 | 21 | 8 | 26 | 4 | 0.7966 | 0.8667 | 0.7241 | 0.7647 |
| ΔP_0_ | 29/30 | 21 | 8 | 24 | 6 | 0.7627 | 0.8000 | 0.7251 | 0.7500 |
| GWS | 29/30 | 23 | 6 | 21 | 9 | 0.7458 | 0.7000 | 0.7931 | 0.7777 |
| ***Maj*** | **29/30** | **28** | **1** | **27** | **3** | **0.9322** | **0.9000** | **0.9655** | **0.9642** |

*Table S2 Results of classification based on individual features extracted from the leader-follower data from the avatar experiment, 30 patients and 29 controls. Features: – profile of relative phase during interaction; – distribution of absolute phase lag over frequencies; Maj – majority over and .*

| Feature | Ctrls/Pts | TN | FP | TP | FN | Accuracy | Sensitivity | Specificity | Precision |
| --- | --- | --- | --- | --- | --- | --- | --- | --- | --- |
| *ϕ_r_(t)* | 29/30 | 26 | 3 | 21 | 9 | 0.7966 | 0.7000 | 0.8966 | 0.8750 |
| |*ϕ_r_(f)|* | 29/30 | 23 | 6 | 27 | 3 | 0.7797 | 0.7667 | 0.7931 | 0.7931 |
| ***Maj*** | **29/30** | **28** | **1** | **17** | **13** | **0.7627** | **0.5667** | **0.9655** | **0.9444** |

*Table S3 Results of classification based on the leader-follower data from the iCub experiment, 21 patients and 21 controls. Features: – profile of relative phase during interaction (majority over 2 sets of coordinates from multidimensional scaling); – distribution of absolute phase lag over frequencies (majority over 14 sets of coordinates from multidimensional scaling); Maj – majority over and (majority over 16 sets of coordinates: 2 sets of coordinates for and 14 sets of coordinates for ).*

| Feature | Ctrls/Pts | TN | FP | TP | FN | Accuracy | Sensitivity | Specificity | Precision |
| --- | --- | --- | --- | --- | --- | --- | --- | --- | --- |
| *ϕ_r_(t)* | 22/22 | 22 | 0 | 12 | 10 | 0.7727 | 0.5455 | 1 | 1 |
| |*ϕ_r_(f)|* | 22/22 | 21 | 1 | 17 | 5 | 0.8636 | 0.7727 | 0.9545 | 0.9444 |
| ***Maj*** | **22/22** | **21** | **1** | **17** | **5** | **0.8636** | **0.7727** | **0.9545** | **0.9444** |

*Table S4 Results of classification based on LSAS anxiety total^1^, autism-spectrum quotient^2^ and conscientiousness from big five inventory^3^ from the clinical evaluation questionnaires collected in the iCub experiment.*

| Data | Ctrls/Pts | TN | FP | TP | FN | Accuracy | Sensitivity | Specificity | Precision |
| --- | --- | --- | --- | --- | --- | --- | --- | --- | --- |
| Questionnaires ^a^ | 22/22 | 20 | 2 | 15 | 7 | 0. 7955 | 0.6818 | 0.9090 | 0.8824 |

a) Clinical indices were rated by experienced clinically trained psychiatrists, blind to the patients’ mirror game performance. The best classification was achieved for the following set of measures (3 out of 10): LSAS anxiety total^1^, autism-spectrum quotient^2^ and conscientiousness from big five inventory^3^ (linear discriminant classifier with leave-one-out validation). Note that, classification based on the neuromotor biomarkers, presented in Table S3 has higher accuracy and precision than classification based on data collected in clinical interviews.

1. Liebowitz, M.R. Social phobia. in *Anxiety. Mod Trends Pharmacopsychiatry.* , Vol. 22 (ed. Klein, D.F.) 141-173 (Karger, Basel, 1987).

2. Baron-Cohen, S., Wheelwright, S., Skinner, R., Martin, J. & Clubley, E. The Autism-Spectrum Quotient (AQ): Evidence from Asperger Syndrome/High-Functioning Autism, Malesand Females, Scientists and Mathematicians. *Journal of Autism & Developmental Disorders* **31**, 5-17 (2001).

3. John, O.P. & Srivastava, S. The Big Five trait taxonomy: History, measurement, and theoretical perspectives. in *Handbook of personality: Theory and research*, Vol. 2 (eds. Pervin, L.A. & John, O.P.) 102-138 (Guilford Press, New York, 1999).

*Note – Stochastic model of a hand motion*

The importance of the shape of movement segments for interpersonal coordination was raised in the recent literature^1,2^. In order to model hand motion in the solo condition of the mirror game (see section *Classification – solo condition*) we used the integrated human movement framework^3^, which consist of three steps:

1. Abstract representation of intended movement (coded as a set of boundary conditions),
2. Generation of virtual minimum-jerk trajectory,
3. Generation of the actual movement according to mass-spring logic.

The model presented in this paper describes steps 1.) and 2.) of the above scheme. The third step is realized by an interactive cognitive architecture described in^4-6^.

The human movement present in the mirror game is complex, non-periodic and specific for each individual person (see Fig. S1). Thus, its generation is a significant challenge. Considering different aspects of this modelling task, we found that using a stochastic process was the best way of capturing its key features.

More specifically, to generate the position trajectory we first produce a sequence of turning-points of position trajectory (that is points where direction of movement changes). To this end we use a Markov process^7^ with coefficients estimated from a pre-recorded trajectory. These turning-points constitute the set of boundary conditions from step 1, which is necessary to generate virtual equilibrium trajectory^3,8,9^ in step 2. The trajectory between the turning points is computed using a 5th order polynomial in accordance with theory of minimum-jerk^3,10-12^. In order to assure that the generated virtual minimum-jerk trajectory is dynamically similar to the source trajectory, we compute the earth’s mover distance between histograms of their velocities^13^.

The algorithm used for generation of the motion has the following structure:

1. Normalise the source position time-series and estimate corresponding velocity and acceleration time-series,
2. Extract coordinates of the turning-points, $p0\left(t\right)=\left\{x(t): v(t)=0\right\}$, by finding times of zero velocity $t_{i}$,
3. Compute lengths of the motion between consecutive turning-points

$\Delta p0\left(t_{i}\right)=\left|p0\left(t_{i}\right)-p0\left(t_{i-1}\right)\right|$,

1. Compute durations of the motion corresponding to the different lengths

$\Delta t\left(t_{i}\right)=\left|t_{i}-t_{i-1}\right|$,

1. Find corresponding values of acceleration at the end of the movements$a_{i}=a_{i}\left(t_{i}\right)$; keep the lengths, $\Delta p0_{i}=\Delta p0\left(t_{i}\right)$, durations $\Delta t_{i}=\Delta t\left(t_{i}\right)$, and accelerations $a_{i}$, as triplets $\left\{\Delta p0_{i},\Delta t_{i},a_{i}\right\}$,
2. Divide the interval [-0.5,0.5] into $N$ bins, where$N$ is one third of the number of turning-points, $N=\#\Delta p0/3$*,*
3. Generate Markov process matrix $\mathbf{L}$ with elements, $L_{jk}=P\left(\left.n_{j}\right|n_{k}\right)$, given by conditional probabilities of being in bin $n_{j}$ at time $t_{i}$conditioned on being in bin $n_{k}$ at time $t_{i-1}$, for $n_{j}>n_{k}$*,* i.e. for movements from left to right,
4. Generate a separate Markov process matrix $\mathbf{R}$ for transition in opposite direction, i.e. for $n_{j}<n_{k}$,

A new trajectory is then generated in the following way:

1. Pick randomly coordinate of the first point $x_{i=1}$,
2. Take matrix $\mathbf{R}$ (or $\mathbf{L}$) and find the next bin. Pick at random from all the coordinates available in the new bin a coordinate of the next turning-point $x_{i+1}$,
3. Compute the length of the movement$\Delta p0_{i}=\left|x_{i+1}-x_{i}\right|$*,* and find the corresponding duration $\Delta t_{i}$ and acceleration at the end of the movement $a_{i+1}$; for the first point assume $a_{1}=0$*,*
4. Use the boundary values:

to find coefficients of the 5^th^ degree polynomial describing single movement segment (using its derivatives):

1. Use the polynomial $p\left(t\right)$ to compute positions between $x_{i}, x_{i+1}$,
2. Increase time by $\Delta t_{i}$,
3. Repeat steps 10 to 14, interchanging matrices $\mathbf{R}$ and $\mathbf{L}$ between repetitions; continue until new trajectory has requested duration.

A check is then carried out to verify if the generated trajectory satisfies similarity condition:

1. Compute earth’s mover distance between velocity profiles of the source and generated trajectories and check if it is smaller than a requested threshold,
2. If the condition is not satisfied repeat steps 9 to 16; otherwise return the generated trajectory.

Figure S1 illustrates the process of extracting information, from the pre-recorded solo trajectory, necessary to generate a new position trajectory with the same dynamical properties as the original one. Fig. S1(A) shows a source position trajectory (black), with the turning points $p0$ indicated with blue dots and lengths of the movement segments $\Delta p0$ indicated by the blue bars. Fig. S1(B) shows the corresponding velocity time-series with the durations of the velocity segments indicated with green bars. Panel (C) shows acceleration trace with the accelerations at the end of the velocity profiles indicated with red dots. Finally, Fig. S1(D) shows an example of a trajectory generated by means of the generative process described above.

To our knowledge we present here the first model for generation of virtual minimum-jerk trajectories in the mirror game. The model can generate human-like motion while preserving motion signature^13^ (style of motion) of individual people. The model can be extended beyond one dimension by means of the latest developments to the minimum-jerk theory^14^.

Fig. S1 Visualisation of the input parameters for the generative process. (A) Position trace with indicated positions at change of direction $p0$ (blue dots) and lengths of movement segment $\Delta p0$ (blue bars). (B) Corresponding velocity trace with durations of velocity segments $\Delta t_{i}$ indicated by the green bars. (C) Acceleration trace with accelerations at the end of a velocity segments $a_{i}$ indicated by red dots. (D) Position trace generated by means of the described generative process fed with parameters from panels (A)-(C).

In order to classify our data we are using two features of our generative process:

- distributions of the lengths of the movement segments $\Delta p0$,
- distributions of the coefficients of the polynomials used to generate all the individual velocity segments of the new generated trajectories:

$v\left(t\right)=c_{5}t^{4}+c_{4}t^{3}+c_{3}t^{2}+c_{2}t+c_{1}$,

However, for classification we are using only coefficients $c_{2}$ to $c_{5}$ because the coefficient $c_{1}=0$ due to the boundary conditions $v\left(0\right)=0, v\left(\Delta t\right)=0$.

Figure S2 illustrates the bivariate distributions of all the pairs of coefficients from the set $\left\{c_{2},c_{3},c_{4},c_{5}\right\}$. Interestingly, the classification produces best results when it is based on all the pairs of coefficients from the set $\left\{c_{2},c_{3},c_{4}\right\}$, what indicates that the bivariate distributions of the coefficients of the lower powers capture some subtle differences between the shapes of velocity segments (a part of the velocity time-series between two consecutive times of zero velocity^15^) of patients and controls.

Fig. S2 Bivariate distributions of the coefficients of the generative process used to generate trajectory in Fig. S1(D).

1. Kilner, J., Hamilton, A.F.d.C. & Blakemore, S.-J. Interference effect of observed human movement on action is due to velocity profile of biological motion. *Social Neuroscience* **2**, 158-166 (2007).

2. Varlet, M.*, et al.* Influence of stimulus velocity profile on rhythmic visuomotor coordination. *J Exp Psychol Hum Percept Perform* **40**, 1849-1860 (2014).

3. Viviani, P. & Flash, T. Minimum-jerk, two-thirds power law, and isochrony: converging approaches to movement planning. *Journal of Experimental Psychology: Human Perception and Performance* **21**, 32 (1995).

4. Zhai, C., Alderisio, F., Tsaneva-Atanasova, K. & di Bernardo, M. Adaptive tracking control of a virtual player in the mirror game. in *53rd IEEE Conference on Decision and Control* 7005-7010 (2014).

5. Zhai, C., Alderisio, F., Tsaneva-Atanasova, K. & di Bernardo, M. A novel cognitive architecture for a human-like virtual player in the mirror game. in *2014 IEEE International Conference on Systems, Man, and Cybernetics (SMC)* 754-759 (2014).

6. Zhai, C., Alderisio, F., Tsaneva-Atanasova, K. & di Bernardo, M. A model predictive approach to control the motion of a virtual player in the mirror game. in *2015 54th IEEE Conference on Decision and Control (CDC)* 3175-3180 (2015).

7. Durrett, R. *Probability: theory and examples*, (Cambridge university press, New York, NY, USA, 2010).

8. Feldman, A.G. Change in the length of the muscle as a consequence of a shift in equilibrium in the muscle-load system. *Biophysics* **19**, 544-548 (1974).

9. Feldman, A.G. Once More on the Equilibrium-Point Hypothesis (λ Model) for Motor Control. *Journal of Motor Behavior* **18**, 17-54 (1986).

10. Flash, T. & Hogan, N. The coordination of arm movements: an experimentally confirmed mathematical model. *The Journal of Neuroscience* **5**, 1688-1703 (1985).

11. Hogan, N. & Flash, T. Moving gracefully: quantitative theories of motor coordination. *Trends in Neurosciences* **10**, 170-174 (1987).

12. Hogan, N. & Sternad, D. On rhythmic and discrete movements: reflections, definitions and implications for motor control. *Experimental Brain Research* **181**, 13-30 (2007).

13. Słowiński, P.*, et al.* Dynamic similarity promotes interpersonal coordination in joint action. *Journal of The Royal Society Interface* **13**, 20151093 (2016).

14. Huh, D. & Sejnowski, T.J. Spectrum of power laws for curved hand movements. *Proceedings of the National Academy of Sciences* **112**, E3950-E3958 (2015).

15. Noy, L., Dekel, E. & Alon, U. The mirror game as a paradigm for studying the dynamics of two people improvising motion together. *Proceedings of the National Academy of Sciences* **108**, 20947-20952 (2011).
